# Insights into the synchronization between DNA replication and parental histone recycling

**DOI:** 10.1042/BST20253014

**Published:** 2025-05-14

**Authors:** Xiaorong Tang, Yuan Yao, Gang Li, Haiyun Gan

**Affiliations:** 1 Shenzhen Key Laboratory of Synthetic Genomics, Guangdong Provincial Key Laboratory of Synthetic Genomics, State Key Laboratory of Quantitative Synthetic Biology, Shenzhen Institute of Synthetic Biology, Shenzhen Institutes of Advanced Technology, Chinese Academy of Sciences, Shenzhen 518055, China; 2 MOE Frontier Science Center for Precision Oncology, Cancer Center, and Department of Biomedical Science, Faculty of Health Sciences, University of Macau, Macau SAR, China

**Keywords:** DNA polymerases, DNA replication, epigenetic inheritance, histone chaperones, parental histones recycling

## Abstract

Accurate parental histone recycling is of pivotal importance in epigenetic inheritance. Its proper functioning hinges on the precise co-ordination among a diverse array of proteins. During DNA replication, any aberration in the distribution of parental histones can potentially result in the loss of epigenetic memory. To date, although several key proteins involved in parental histone recycling have been identified, the detailed molecular mechanisms underlying their functions remain elusive. This mini-review focuses on summarizing the synchrony between DNA replication and parental histone recycling, along with the key participants in parental histone recycling. In the end, we provide an overview of the inherent connection between parental histone recycling and epigenetic inheritance, shedding light on the fundamental role of histone recycling in maintaining epigenetic information across cell divisions.

## Introduction

Nucleosome, the basic unit of chromatin, is composed of 146 base pairs of DNA enwrapped around histone H3-H4 and H2A-H2B complexes [1]. The assembly of nucleosomes proceeds with the deposition of H3-H4 onto DNA first, followed by the addition of H2A-H2B heterodimers. Conversely, the disassembly process occurs in the reverse order [2]. The histone tails are subject to various modifications, which serve as crucial regulatory elements in DNA transactions, including replication, repair, and transcription [3]. During DNA replication, the semi-conservative replication mechanism allows for the retention and transfer of parental histones to the nascent DNA strands. The precise recovery of parental histones plays a pivotal role in maintaining mitotic inheritance and cell fate.

After DNA replication, the old (H3-H4)₂ tetramers usually do not mix with the new H3-H4. However, both the old and new H2A-H2B dimers will mix with the old and new H3-H4 and assemble within the same nucleosome [4]. At the replication fork, the parental histones first disassemble from the octamer into one H2A-H2B proximal–(H3-H4)₂ hexamer and one H2A-H2B distal dimer. The removal of the distal H2A-H2B dimer might be a critical step in histone recycling, facilitating the binding of the Mcm2 histone-binding domain to the histone hexamer. This arrangement also ensures the proper docking of the FACT–histone complex with the fork protection complex subunit Tof1 [5]. *In vitro* experiments show that the removal of Tof1, which works as a scaffold in the replisome, changes the dynamics of histone recycling. The positions of histones are relatively conserved before and after recycling, and they tend to localize on DNA sequences that are energetically stable and favorable for nucleosome formation. However, after removing Tof1, the histone recycling positions change, shifting to DNA sequences that are unfavorable for nucleosome formation [6].

The allocation mechanism of parental histone H3-H4 is highly conserved in mammals and budding yeast [7–9]. The mini-chromosome maintenance (MCM) helicase subunit Mcm2 [10] deposits parental histone modifications onto the lagging strands. When Mcm2 is mutated, parental histone recycling presents an unbalanced distribution [11]. Through a combination of molecular dynamics simulations and *in vitro* experiments, it has been found that Mcm2 can directly deposit H3-H4 onto the daughter strands without the assistance of histone chaperones [12]. Meanwhile, Dpb3/4 [13] guides the allocation of parental histones to the leading strands [8,14,15]. Mutations in either Mcm2 or Dpb4 can lead to a strand preference in the parental histone recycling. However, this bias is reduced in cells with double mutations in Mcm2 and Dpb4, approaching the strand bias of wildtype cells [15,16], suggesting that the parental histone recycling for the leading and lagging strands might be mutually independent. Moreover, the parental H2A-H2B histones can be symmetrically distributed onto the two daughter strands, and the allocation mechanism is independent of the parental histone recycling pathway for H3-H4 and is not mediated by POLE4/Dpb3 or Mcm2. In the absence of transcriptional re-initiation, the modifications of the parental H2A-H2B histones can also be accurately and rapidly restored [17].

Through the real-time single-molecule visualization of replication fork to capture the dynamics of the replication fork, it was discovered that the majority of parental histones are evicted from the DNA, which represents the primary consequence of the encounter between the replication fork and nucleosomes. However, when newly synthesized histones are depleted, local histone recycling becomes predominant, indicating that the concentration of free histones is a crucial regulator of the dynamic changes in parental histones at the replication fork [18]. Varying levels of accessible free histones, based on the distinct characteristics of chromatin regions, can inhibit the local histone recycling in euchromatin regions; conversely, they contribute to promoting histone recycling in heterochromatin regions [18].

In this mini-review, after describing the basic details of parental histone recycling, we focus on summarizing the connection between DNA replication and histone recycling and propose the concept of DNA replication–parental histone recycling synchronization. Then we outline the participants in parental histone recycling. What’s more, based on extended research, we summarize the practical implications of parental histone recycling in biological processes.

### DNA replication–parental histone recycling synchronization

Parental histone recycling is closely intertwined with DNA replication. During DNA replication, the double-stranded DNA unwinds and new DNA strands are synthesized. Concurrently, the parental histones associated with the original DNA need to be recycled and reassembled onto the newly synthesized DNA strands. This process is crucial for maintaining epigenetic information carried by histones, such as histone modifications, which play vital roles in gene regulation.

**Figure 1:**
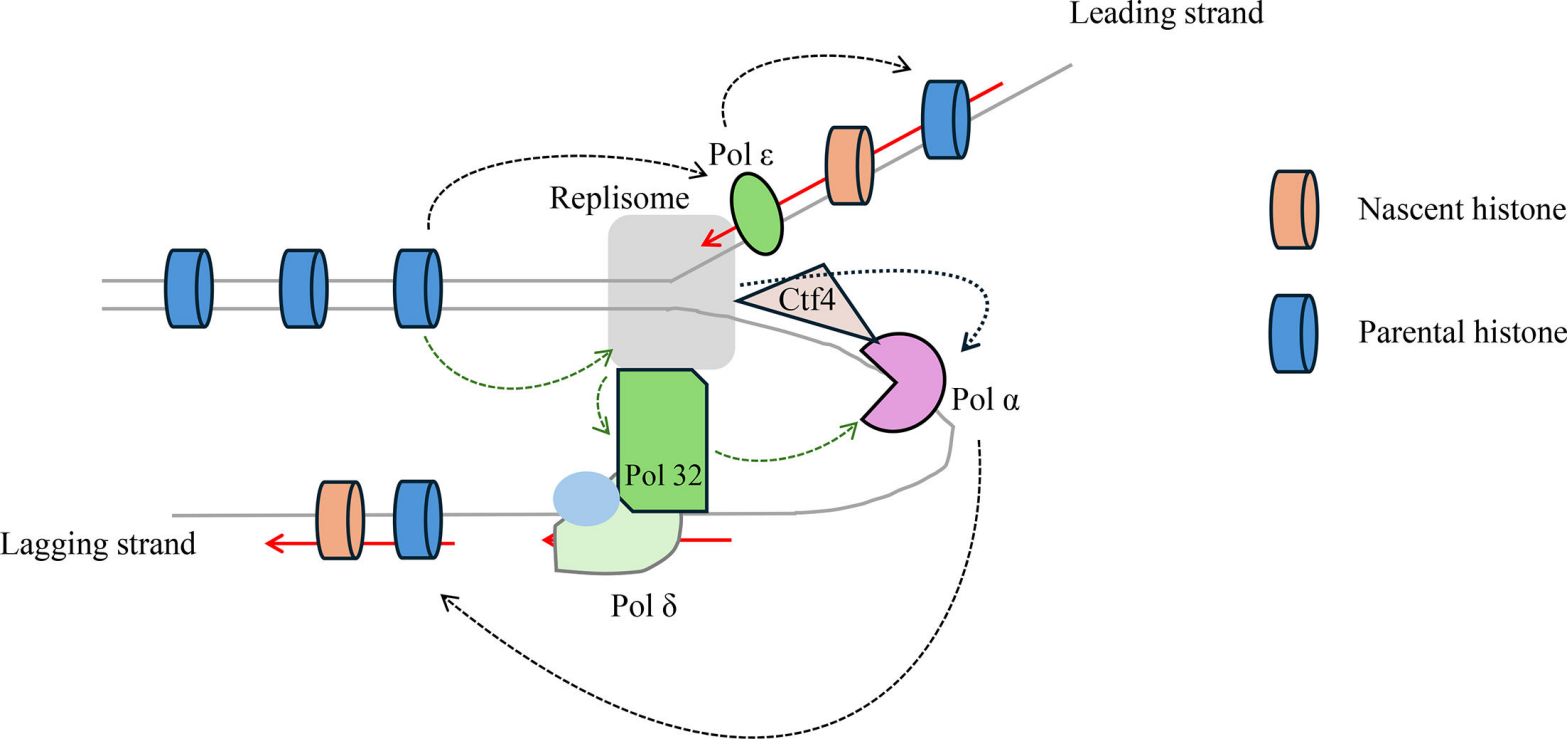
A model for the role of DNA polymerases in parental histone transfer. DNA polymerase epsilon is responsible for depositing parental histones on the leading strand. DNA polymerase alpha (Pol α) binds to Mcm2 via the Ctf4 adaptor protein. This interaction allows Pol α to transfer parental histones to the lagging strand DNA. Pol32, a non-essential subunit of DNA polymerase delta, can interact with Mcm2 independently of Ctf4 and transfer parental histones to the lagging strand.

DNA polymerases are key enzymes in DNA replication. Different DNA polymerases have distinct functions. For example, DNA polymerase alpha (Pol α) is responsible for initiating DNA synthesis after the RNA primer. It provides a crucial foundation for recruiting other replication-related proteins and subsequent DNA strand elongation [19,20]. Moreover, polymerase epsilon (Pol ε) elongates the leading strand [21,22], while polymerase delta (Pol δ) replicates the lagging strand [23]. With the deepening of research, an increasing number of findings have been successively reported the direct or indirect roles of DNA polymerases in parental histone recycling. These discoveries have further elucidated the close and synchronous relationship between histone recycling and DNA replication (Figure 1).

As described above, Dpb4/Dpb3, which are subunits of Pol ε [13], are widely studied for their role in recycling parental histones H3-H4 to the leading strand [12]. In the absence of Dpb4 or Dpb3, the distribution of parental histones shows a preference for the lagging strand [8,15,16].

DNA Pol α, another important polymerase, binds to Mcm2 via the Ctf4 adaptor protein. This interaction enables Pol α to transfer parental H3-H4 histones to the lagging strand DNA [9,24]. In addition, Pol α can also bind to histone H2A-H2B [25] and recycle the parental H2A-H2B to the lagging strand [17].This indicates that Pol α serves as a docking platform not only for the parental H3-H4 but also for H2A-H2B.

DNA Pol δ, with its non-essential subunit Pol32, is mainly responsible for the lagging strand DNA synthesis. Pol32 not only plays an indispensable role in nucleosome assembly but also is essential for the regulation of Okazaki fragment processing. It bridges nucleosome assembly and Okazaki fragment processing, allowing these two seemingly independent processes to co-ordinate and co-operate with each other during DNA replication through the function of Pol32 [26]. Pol32 has the ability to bind to histones H3-H4 [26], which depends on the histone-binding function of Mcm2, allows for the transfer of parental H3-H4 to the lagging strand. A connection, Mcm2-Pol32-Pol1 (Pol α catalytic subunit), has been discovered [27]. Independent of the replisome hub protein Ctf4, Pol32 interacts with Mcm2 and receives the parental H3-H4 bound by Mcm2 and transfers them to the lagging strand. In addition, Pol δ may not participate in the allocation of parental histones to the lagging strand through Mcm2. Since proliferating cell nuclear antigen (PCNA) tethers the DNA polymerases to DNA, when PCNA loses its binding ability to Pol32, the recycling of parental histones will be impaired [28].

In summary, parental histone recycling and DNA replication are synchronous processes, and DNA polymerases act as crucial mediators in this complex molecular mechanism, facilitating the accurate transfer of genetic and epigenetic information.

### Other non-replisome histone chaperones

Throughout their entire life cycle, histones are invariably accompanied by histone chaperones, which actively participate in a diverse array of activities, including transportation, degradation, and chromatin dynamics. Parental histone recycling, as a crucial step in DNA replication, necessitates the co-ordinated actions of multiple chaperone factors. The employment of these accessory factors may endow cells with the ability to transform chromatin states. Notably, during DNA replication, histones on both the leading and lagging strands rely on common *in vivo* chaperones. Prominent examples of such chaperones are Chromatin Assembly Factor 1 (CAF-1), Anti Silencing Function 1 (ASF1), and Facilitates Chromatin Transcription (FACT).

CAF-1 was discovered in 1989 [29]. In budding yeast, it consists of three subunits: Cac1, Cac2, and Cac3. Among them, Cac1 serves as a scaffold for CAF-1 to bind to H3-H4 [30]. CAF-1 can facilitate the right-handed wrapping of DNA around the H3-H4 histones [31]. During DNA replication, the new histones deposited by CAF-1 create a chromatin environment conducive to sister chromatid cohesion and the maintenance of genomic integrity. Once CAF-1 is absent, the number of nucleosomes deposited on the replicated DNA will decrease [32]. In mouse embryonic stem cells (mESCs), it has been found that CAF-1 deposits newly synthesized histones on both the leading and lagging strands. However, the recycling of parental histones does not depend on CAF-1. When CAF-1 is removed, the parental histones can still be distributed nearly symmetrically on the two daughter strands [33].

ASF1 is a highly conserved histone chaperone in eukaryotes. It mainly participates in the assembly and disassembly of nucleosomes, and the exertion of its functions depends on its interaction with H3 and H4 [34]. During DNA replication, ASF1 can mediate the deposition of H3K56 acetylation, thereby playing a role in stabilizing the genome [35]. Nevertheless, in the filamentous fungus *Sordaria macrospora*, the function of ASF1 in maintaining genome stability does not rely on its histone-binding ability. Even when its histone-binding ability is removed *in vivo*, ASF1 can still facilitate the protection against DNA damage [36]. ASF1 can promote the recycling of parental histones, but its specific mechanism remains unclear currently. What is known is that when ASF1 is absent, the parental histones become more dispersed after DNA replication [37]. When DNA replication stalls, the amount of ASF1 accumulated in the nucleus increases, and histones with post-translational modifications will be retained in the ASF1 complex. Among them, the content of H4K5K12diAc (a marker of new histones) bound to ASF1 increases, indicating that ASF1 can transfer newly synthesized histones during the replication process. Meanwhile, after replication is restarted, the number of interacting modified histones in the ASF1 complex decreases significantly, further supporting the role that ASF1 plays in the recycling of parental histones [38,39].

FACT [40] is highly conserved and can interact with nucleosomes and histone H2A/H2B heterodimers [41]. The FACT complex co-operates with Mcm2 and Dpb3/4 to achieve the allocation of parental histones and the heterochromatin inheritance [15]. The replisome component Mcl1 in *Schizosaccharomyces pombe* (an ortholog of *Saccharomyces cerevisiae* Ctf4 and mammalian AND1) interacts with Mcm2 and FACT, assisting FACT in the transfer of parental histones [42]. FACT maintains the allocation of parental histones on both strands during DNA replication. Moreover, FACT can also regulate the inheritance of parental histones through replication-independent turnover [37]. In *S. cerevisiae*,the depletion or mutation of the spt16 subunit of FACT can disrupt histone binding, thereby affecting the recycling of parental histones on both the leading and lagging strands [43]. Spt16 is involved in both the deposition of newly synthesized histones and the transfer of old histones, and its N-terminal domain functions as a protein interaction module. The depletion of Spt16 weakens the interaction between FACT and MCM2-7, thus influencing the recycling of parental histones, but it does not affect the binding of FACT or MCM2-7 to chromatin. Moreover, the effect of spt16-N on the transfer of parental histones to the lagging strand is more pronounced [43]. The mutation of FACT reduced the incorporation of H2A/H2B into nucleosomes in the heterochromatin region [44]. FACT can also promote the spread of heterochromatin by inhibiting histone turnover in the heterochromatin region [45].

The recycling of parental histones is also regulated by the components of the replisome with histone-binding properties. Mrc1, a subunit of the fork protection complex, collaborates with Mcm2 to assist in the allocation of parental histones to the lagging strand during DNA replication [46]. Interestingly, Mrc1 may be capable to toggle histones between the two DNA strands. Mutations in the connector domain of Mrc1 disrupts histone recycling on the lagging strand, while another mutation related to histone binding impairs the recycling of histones on the leading strand [46,47]. During the allocation of parental histones to daughter strands, Mrc1 might serve as an overlapping allocation site, mimicking the characteristics of a nucleosome and forming a scaffold around H3-H4 [47].

### The biological functions of parental histone recycling

During DNA replication, the correct and timely transfer of parental histones is crucial for maintaining the structure and function of chromatin. Impairment in the recycling of parental histones leads to a significant reduction in the frequency of homologous recombination within cells, which exerts a detrimental effect on the cells [16,48]. Mutations occurring in the Mcm2–Ctf4–Pol α and Dpb3–Dpb4 pathways lead to a decrease in error-free DNA damage tolerance, increasing cellular mutations and drug resistance [49]. The mutation of Mcm2 and Dpb3/Dpb4 results in abnormal chromosome segregation [50] and causes a higher silencing-loss rate. This indicates that defects in the replisome have an impact on epigenetic inheritance, while having relatively little influence on heterochromatin stability [51]. Furthermore, the mutations in Mcm2 and Dpb3/Dpb4 are responsible for causing a bias in the distribution of parental histones onto the daughter strands. It is highly plausible to postulate that the dysregulation of parental histones might also be a contributing factor to the loss of epigenetic silencing.

During DNA replication, parental histone recycling, as one of the key steps, involves the co-ordinated action of numerous proteins. For instance, the various polymerase subunits previously mentioned, such as Pol α, Pol32, along with replication-related protein complexes, all contribute to the precise positioning of parental histones at appropriate loci. Notably, these proteins also play a crucial role in epigenetic memory, implicitly indicating a close association between parental histone recycling and epigenetic memory. Once an imbalance occurs in the allocation of parental histones, it is highly probable to result in the loss of epigenetic memory, thereby exerting a profound impact on many aspects, including the normal physiological functions of cells and the transmission of genetic information.

FACT, Mcm2, and Dpb4/3 may have redundant functions inheterochromatin inheritance. FACT is recruited to the heterochromatin region, where it plays a role in maintaining silencing [44]. Additionally, it can facilitate heterochromatin spreading by inhibiting histone turnover within this region [45]. Mcm2 affects histone recycling and heterochromatin silencing via its histone binding function. The replisome component Mcl1/Ctf4 promotes the interaction between FACT and Mcm2. In the event that Mcm2 loses its histone binding function, Mcl1 can still assist FACT in retaining parental methylated histones and propagate this methylation modification through a read-write mechanism. During the process of heterochromatin propagation, Mcl1 may be a more important regulator than Mcm2 [42]. Mrc1, which is one of the fork protection complex subunits, collaborates synergistically with Mcm2 to aid in the allocation of parental histones to the lagging strand. Mrc1 can stabilize heterochromatin solely based on its function in genomic stability [46].

Currently, there is a general consensus that chromatin-based epigenetic memory relies on the accurate distribution of modified parental histones onto newly synthesized DNA strands. The central question is how these modified parental histones are distributed, and which factors exert an influence on this process. For example, the speed of the replication fork, the concentration of free histones [18], and the accessibility of histone chaperones in distinct chromatin regions may all play a role in regulating their recycling rates. Although it was believed that parental histones were recycled to the nascent strands symmetrically [6,11], contemporary research reveals that this distribution is not entirely symmetrical. In mESCs, parental H3K9me3 distribution displays a bias toward leading strands and mainly occurs at long interspersed nuclear element. This process is mediated by the interaction between the human silencing hub and Pol ε [52]. Nevertheless, the underlying mechanisms remain elusive. The reasons why such a preference exists in retrotransposons and what biological implications it entails are questions that still demand in-depth investigation.

The positional information of post-translational histone modifications can be preserved through parental histone recycling. Histone modifications can be rapidly and accurately restored on newly synthesized DNA, following marker and position-specific kinetics [53]. In *S. cerevisiae*, parental histones can be recycled to the heterochromatin boundaries of the daughter DNA strands and their positional information can be restored. This indicates that parental histones can be recycled to their original positions, albeit not with absolute precision [37]. When the synthesis of new histones is deficient and only parental histones are available in the cells, the modifications of parental H2A-H2B and H3 are largely restored across the genome, suggesting that parental histones are recycled *in vivo* to preserve the epigenetic landscape [54].

In fact, the rate of parental histone recycling may not be uniform during DNA replication. At genomic locations where the H2AK119ub1 modification is pre-enriched, the recovery speed of H2AK119ub1 is the fastest [17]. Similarly, the restoration of H3K27me3 shows position specificity. For instance, it is evident at the sites where the Polycomb Repressive Complex 2 is enriched [53] or within the heterochromatin regions [55]. Moreover, the parental H3K27me3 domains remain in a relatively stable state throughout the cell cycle [53]. In addition, there may be a certain correlation between the recycling of parental H2A-H2B histones and that of H3-H4 histones. Notably, H2AK119ub1 can also guide the accurate restoration of H3K27me3 [17]. Heterochromatin inheritance is related to both the symmetrical distribution of parental H3-H4 histones and the density of parental H3-H4 histones [15].

The classic model posits that the marked histones carry epigenetic information. Upon DNA replication, the modifications of the marked parental histones are recycled to their original positions, and histone modifiers are recruited to assist in the deposition of new marks on adjacent nucleosomes, thereby reconstructing the histone modification landscape [17,37]. Nevertheless, research conducted by Saxton and Rine challenges this perspective [51]. Investigations have revealed that even when the balance of parental H3-H4 recycling is disrupted and the number of parental H3-H4 tetramers is reduced, there is no significant correlation between the silencing loss rate in the heterochromatin region and the number of nucleosomes. The recycling of H3-H4 tetramers has the minimal impact on the inheritance of the silenced state. These findings imply that histone H3-H4 tetramers may not carry the memory of the silenced state during DNA replication [51].

All in all, although the mechanism by which histone modifications carry and transmit epigenetic information remains elusive,, the crucial role that parental histone recycling plays in determining cell fate is beyond doubt. Once disorders occur in the parental histone recycling process, they will lead to changes in chromatin structure and gene expression profiles, subsequently triggering abnormalities in neural development and embryonic development [7,56].

As one of the key research frontiers in the field of epigenetics, the parental histone recycling mechanism has attained remarkable phased achievements in recent years. However, there still exists a vast space for exploration in this field. For example, the conservation and specificity of parental histone recycling among different species require further enhancement and in-depth exploration in relevant research. In the future, it is expected that, aided by advanced techniques such as cryo-electron microscopy and single-molecule fluorescence imaging, the high-resolution structures of the dynamic binding of various factors with parental histones during the progression of the replication fork can be analyzed. This will enable the construction of a more precise molecular interaction model and the elucidation of the spatiotemporal-specific regulatory network of histone recycling.

**Perspectives**

- Due to the important role of parental histone recycling in maintaining epigenetic genome stability, ensuring the accurate transmission of genetic information, and affecting cell fate, in-depth research on the mechanism of parental histone recycling will be helpful to better understand the basic processes of life activities and provide new ideas and methods for the treatment of related diseases.
- After describing the basic details of parental histone recycling, we focus on summarizing the connection between DNA replication and histone recycling and propose the concept of DNA replication–parental histone recycling synchronization. Then we outline the participants in parental histone recycling. What’s more, based on extended research, we summarize the practical implications of parental histone recycling in biological processes.
- Research on the cross-species mechanisms in parental histone recycling will promote our understanding of its pivotal role in disease development. Ultimately, these outcomes will contribute substantially to human health research, potentially leading to the discovery of novel diagnostic markers, therapeutic targets, and preventive strategies for various diseases, thereby improving the overall well-being of humanity.

## Abbreviations

ASF1: Anti Silencing Function 1
CAF-1: Chromatin Assembly Factor 1
FACT: Facilitates Chromatin Transcription
PCNA: proliferating cell nuclear antigen
Pol α: polymerase alpha
Pol δ: polymerase delta
Pol ε: polymerase epsilon
mESC: mouse embryonic stem cells
